# Got spirit? The spiritual climate scale, psychometric properties, benchmarking data and future directions

**DOI:** 10.1186/s12913-017-2050-5

**Published:** 2017-02-11

**Authors:** Keith Doram, Whitney Chadwick, Joni Bokovoy, Jochen Profit, Janel D. Sexton, J. Bryan Sexton

**Affiliations:** 1 Adventist Health, Palo Alto, CA USA; 2 Department of Pediatrics, Stanford University School of Medicine and Lucile Packard Children’s Hospital, Palo Alto, CA USA; 3 Perinatal Epidemiology and Health Outcomes Research Unit, Division of Neonatology, Department of Pediatrics, Stanford University School of Medicine and Lucile Packard Children’s Hospital, Palo Alto, CA USA; 4 Duke Patient Safety Center, Duke University Health System, Durham, NC USA; 5 Department of Psychiatry, Duke University School of Medicine, Duke University Health System, Durham, NC USA; 6 Residency Office MC 5906, Lucile Packard Children’s Hospital Room 0111, 725 Welch Rd, Palo Alto, CA 94304 USA

**Keywords:** Spirituality, Spiritual climate, Workplace spirituality, Safety climate, Safety culture, Survey, Scale

## Abstract

**Background:**

Organizations that encourage the respectful expression of diverse spiritual views have higher productivity and performance, and support employees with greater organizational commitment and job satisfaction. Within healthcare, there is a paucity of studies which define or intervene on the spiritual needs of healthcare workers, or examine the effects of a pro-spirituality environment on teamwork and patient safety. Our objective was to describe a novel survey scale for evaluating spiritual climate in healthcare workers, evaluate its psychometric properties, provide benchmarking data from a large faith-based healthcare system, and investigate relationships between spiritual climate and other predictors of patient safety and job satisfaction.

**Methods:**

Cross-sectional survey study of US healthcare workers within a large, faith-based health system.

**Results:**

Seven thousand nine hundred twenty three of 9199 eligible healthcare workers across 325 clinical areas within 16 hospitals completed our survey in 2009 (86% response rate). The spiritual climate scale exhibited good psychometric properties (internal consistency: Cronbach α = .863). On average 68% (SD 17.7) of respondents of a given clinical area expressed good spiritual climate, although assessments varied widely (14 to 100%). Spiritual climate correlated positively with teamwork climate (*r* = .434, *p* < .001) and safety climate (*r* = .489, *p* < .001). Healthcare workers reporting good spiritual climate were less likely to have intentions to leave, to be burned out, or to experience disruptive behaviors in their unit and more likely to have participated in executive rounding (*p* < .001 for each variable).

**Conclusions:**

The spiritual climate scale exhibits good psychometric properties, elicits results that vary widely by clinical area, and aligns well with other culture constructs that have been found to correlate with clinical and organizational outcomes.

## Background

Against the backdrop of a struggling economy healthcare workers care for more and sicker patients, while coping with a rapidly changing and increasingly high-tech environment requiring healthcare workers to interact more with screens instead of people. During these times of high burnout and low engagement, [1] health care workers want to “bring their whole selves” to work, through recognition and acceptance of their spirituality [2]. Research on spirituality in the workplace expanded after Mitroff and Denton [2], demonstrated the benefits of assessing “spirituality” over “religion”. Whereas prior research had focused almost exclusively on religious *affiliations, practices and values*, subsequent studies were primarily concerned with the *personal meanings* that people attached to spirituality. Workplace spirituality was defined by Ashmos and Duchon [3], as “the recognition that employees have an inner life that nourishes and is nourished by meaningful work that takes place in the context of community” [4]. Organizations that embrace workplace spirituality by encouraging the respectful expression of diverse spiritual views have staff who report bringing more of their “complete selves” to work [2, 5]. Work environments, both within and outside of the healthcare industry, with superior spiritual climates have higher productivity and performance and support employees with greater emotional intelligence, organizational commitment and job satisfaction when compared to their lesser counterparts [3, 5–7]. Despite these encouraging findings, taboos and misperceptions often obstruct the utility of workplace spirituality in healthcare. Most workers adamantly report desires to discuss and express their spirituality in the workplace, but they are hesitant to do so for fear of offending their peers [8, 9]. The majority of workers believe that their coworkers are uncomfortable discussing spirituality, while in fact the opposite is true [9]. Given the positive effect of promoting spiritual climate in other industries and the unique burdens of the health care environment, challenging these longstanding behavioral norms may prove beneficial to health care workers and patients. Anecdotally, we are frequently asked by hospitals to “please give them a sense of spiritual climate” by incorporating their local assessments of workplace spirituality into debriefings of employee engagement and safety culture results using their home-grown or published workplace spirituality metrics. Unfortunately, these well-intentioned spirituality assessments often lack the rigor needed for them to be useful in strategic planning due to very low response rates and lack of representation across all the clinical areas. A metric that simultaneously provided a snapshot of spiritual climate and identified clinical areas in need of a “deeper-dive” with a more comprehensive tool could be quite useful. Survey fatigue is widespread in healthcare, so we sought a very brief metric of “a sense of spiritual climate” that could be used across all clinical areas as part of a routine employee engagement or safety culture survey administration. To date, there is no widely used definition of spirituality. Researchers are still trying to define basic terms and standards for interpretation, and existing metrics tend to be lengthy and multidimensional. Our aim was to generate a simple brief metric of spiritual climate that could capture the extent to which spirituality is accepted within a clinical area, signaling the utility of subsequent use of a more comprehensive assessment of spirituality. Here we describe the reliability, validity, and initial benchmarking data of a novel brief measure of spiritual climate for use in healthcare work environments.

## Methods

### Design and study population

This is a cross-sectional study of archival survey data collected in 2009 from 7923 healthcare workers across 325 clinical areas within 16 hospitals of a faith-based health system on the West Coast. Data were subsequently shared with JBS for retrospective analysis. Scale validation was not part of the 2009 organizational assessment of safety culture. All staff with a 50% or greater commitment to their patient care area for at least the 4 consecutive weeks prior to survey administration were invited to complete the questionnaire regardless of their involvement in patient safety endeavors. This included staff physicians, registered nurses (RN), charge nurses, nurse managers, physician assistant/nurse practitioners, licensed vocational nurses (LVN)/licensed practicing nurses (LPN), hospital aides, physical therapists, occupational therapists, pharmacists, respiratory therapists, technicians, ward clerks/unit secretaries, medical administrators, and others. Demographic data for the entire sample is presented in Table 1. All clinical areas within each hospital and its affiliated ambulatory clinics were asked to participate. Surveys were administered and collected during pre-existing departmental and staff meetings. The survey distributed to healthcare workers was comprised of demographic items, the Safety Attitudes Questionnaire (SAQ), [10] intention to leave items, burnout items, and items pertaining to participation in Executive Rounding (also known as patient safety leadership walkrounds) [11] and five novel spiritual climate items. Together these instruments constituted the “survey” administered across all 16 hospitals.

**Table 1:** Respondent Demographics and Cronbach’s α by Caregiver Type

		*N*	Cronbach’s α	% of Total
Caregiver Type Name	Registered Nurse	2797	.867	35.3%
	Technologist/Technician (e.g., Surg., Lab, Rad)	1036	.852	13.1%
	Clinical Support (CMA, EMT, Nurses Aide, etc.)	890	.853	11.2%
	Admin Support (Clerk/Secretary/Receptionist)	664	.846	8.4%
	Therapist (RT, PT, OT, Speech)	555	.869	7.0%
	Other	455	.875	5.7%
	Nurse Manager/Charge Nurse	318	.849	4.0%
	LVN/LPN	233	.808	2.9%
	Attending/Staff Physician	230	.859	2.9%
	Other Manager (e.g., Clinic Manager)	110	.835	1.4%
	Pharmacist	101	.882	1.3%
	Resident Physician	54	.882	.7%
	Physician Assistant/Nurse Practitioner	52	.914	.7%
	Clinical Social Worker	48	.913	.6%
	Environmental Support (Housekeeper)	42	.865	.5%
	Dietician/Nutritionist	41	.893	.5%
	Fellow Physician	4	.860	.1%
	Missing	293	.868	3.7%
Gender	Male	1658		20.9%
	Female	5906		74.5%
	Missing	359		4.5%
Shift	Days	4648		58.7%
	Evenings	415		5.2%
	Nights	1303		16.4%
	Variable Shifts	705		8.9%
	Missing	852		10.8%
Years in Specialty	less than 6 months	293		3.7%
	6–11 months	345		4.4%
	1–2 years	1022		12.9%
	3–4 years	997		12.6%
	5–10 years	1701		21.5%
	11–20 years	1618		20.4%
	21 or more years	1530		19.3%
	Missing	417		5.3%
	Total	7923		

### Measurements

The spiritual climate scale was developed by JDS and JBS. The final version of the scale contains 4 items:

- I am encouraged to express spirituality in this clinical area.
- My spiritual views are respected in this clinical area.
- My spirituality has a comfortable home in this clinical area.
- A diverse set of spiritual views are accepted in this clinical area.

The response scale for the spiritual climate items ranges from 1 (disagree strongly) to 5 (agree strongly). Originally 5 items, a fifth item “People in this clinical area are comfortable talking about God” was dropped from the scale following feedback from chaplains who felt inclusion of the word “God” in an item might exclude individuals whose spirituality or religion does not include a single creator or deity.

### Statistical analysis

We used reliability analyses to evaluate the 4-item spiritual climate scale. Internal reliability was assessed using Cronbach’s α. Using ANOVA, we tested for differences on the spiritual climate scale score by hospital, clinical area, and healthcare worker role. Spiritual climate scale scores were computed by taking the mean of the four items, subtracting 1, and then multiplying by 25 for a score which would range from 0 to 100. In addition to the means, we also report the percent agreement (agree slightly plus agree strongly) for items and scale scores of each healthcare worker role and hospital. We call this ‘percentage agree’ or ‘percentage reporting good spiritual climate.’ In exploratory analyses to put spiritual climate into context, we correlated mean spiritual climate scores with mean teamwork and safety climate scores, and several other available variables of interest from the same survey, namely participation in executive rounding and turnover intention, burnout, and disruptive behavior using exploratory two-tailed Pearson correlations. Effects sizes were calculated using partial eta squared (0–.1 weak, .1–.3 modest, .3–.5 is moderate and > .5 is strong) and Cohen’s d (effect size thresholds of small (0.2), medium (0.5) and large (0.8) are used). All statistical analyses were performed using IBM SPSS version 21.

### Ethics statement

This study was determined to be exempt from review by the Institutional Review Board at Duke University, Durham, North Carolina.

## Results

### Respondent demographics

There were 7923 surveys returned from the 16 hospitals studied. Overall response rate was 86% (7923 out of 9199). Registered nurses accounted for 35% of responses (*n* = 2797), technicians 13% (*n* = 1036), clinical support 11% (*n* = 890), administrative support 8% (*n* = 664), therapists 7% (*n* = 555), nurse managers 4% (*n* = 318), LVN/LPNs 3% (233), attending physicians 3% (*n* = 230), other managers 1.4% (*n* = 110) and pharmacists 1.3% (*n* = 101). Less than 1% of the sample was comprised of resident physicians (*n* = 54), physician assistants/nurse practitioners (*n* = 52), social workers (*n* = 48), environmental support (*n* = 42), nutritionists (*n* = 41) and fellows (*n* = 4). 10% of respondents did not identify with one of the listed healthcare worker roles. Respondents were predominantly female (74%) and day-shift workers (59%), with diversity in years of experience in their specialty. See Table 1 for an additional breakdown of respondent demographics.

### Spiritual climate scale internal reliability and correlations with teamwork climate, safety climate, intention to leave, burnout, and disruptive behaviors

**Fig. 1:**
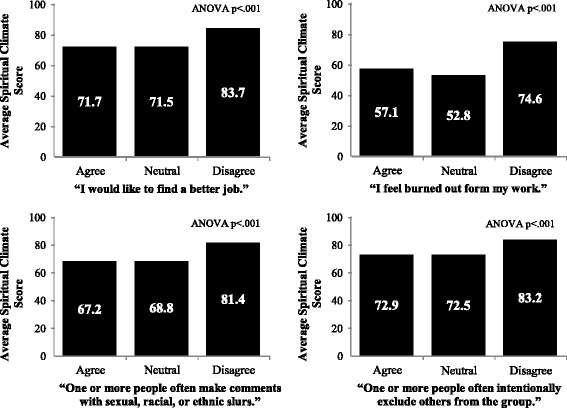
Spiritual Climate and Intention to Leave; Spiritual Climate and Burnout (Emotional Exhaustion)

Analysis of all responses confirmed a high degree of internal consistency with an overall α = .86. By hospital, spiritual climate internal consistency ranged from α = .82 to α = .88, and by position it ranged from α = .81 to α = .91 (Table 1). Pearson Correlation of spiritual climate with teamwork climate was *r* = .43, *p* < .001; and with safety climate it was *r* = .49, *p* < .001. Pearson Correlation of spiritual climate with “I would like to find a better job,” was *r* = −.28, *p* < .001, and with “I feel burned out from my work,” was *r* = −.24, *p* < .001 (Fig. 1). The Pearson correlation of spiritual climate with disruptive behaviors items: in this clinical area, one or more people often: “intentionally exclude others from the group,” was *r* = −.25, *p* < .001, and “make comments with sexual, racial, or ethnic slurs,” was *r* = −.24, *p* < .001 (Fig. 1).

### Variation in spiritual climate by healthcare worker role, hospital and clinical area

**Fig. 2:**
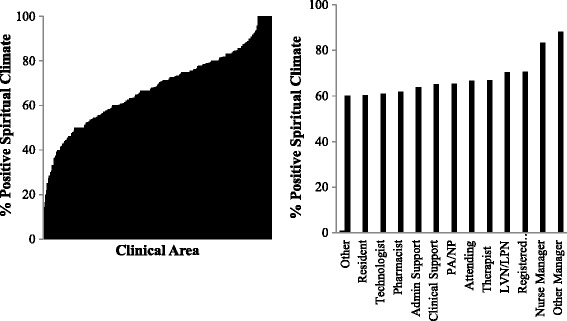
Spiritual Climate by Clinical Area and Healthcare Worker Role

Univariate ANOVA demonstrated significant differences in spiritual climate scale scores between healthcare worker roles *F* (17, 7747) = 8.69, *p* < .001, ηp2 = 0.019, hospitals *F* (15, 7747) = 10.27, *p* < .001, ηp2 = 0.020 and clinical areas *F* (324, 7747) = 3.06, *p* < .001, ηp2 = 0.118 (Fig. 2).

### Associations with quality, outcomes and interventions: clinical areas in the top and bottom quartiles of spiritual climate

The mean spiritual climate scale score was *M* = 67.77, *SD* = 18.10. Clinical areas in the bottom quartile ranged from 14.3 to 55.6% reporting positive spiritual climate, *M* = 43.88, *SD* = 11.14, and those in the top quartile ranged 80 to 100% positive spiritual climate, *M* = 88.78, *SD* = 7.27. Independent-samples t-tests indicated that top and bottom quartile spiritual climate scores were associated with significant differences in teamwork climate *t*(162) = −7.63, *p* < .001, *d* = 1.11, safety climate *t*(162) = −9.88, *p* < .001, *d* = 1.30, exclusionary disruptive behaviors *t*(162) = 5.31, *p* < .001, *d* = −.65, sexual/racist/ethnic slur disruptive behaviors *t*(162) = 4.72, *p* < .001, *d* = −.30, burnout *t*(162) = −3.18, *p* = .002, *d* = −.51, intentions to leave *t*(162) = 2.99, *p* = .003, *d* = −.31, participation in patient safety leadership WalkRounds *t*(162) = −4.50, *p* < .001, *d* = .60 and receiving feedback about WalkRounds *t*(162) = −5.84, *p* < .001, *d* = .86 (Table 2).

**Table 2:** Top vs. bottom spiritual climate quartiles with *t*-values, *p* level, and effect sizes using Cohen’s *d.* A *p*-value of <0.05 is used to determine statistical significance and effect size thresholds of small (0.2), medium (0.5) and large (0.8) are used

Variable	1^st^ spiritual climate quartile M(SD)	4^th^ spiritual climate quartile M(SD)	*t*	*p*	*d*
% reporting good Teamwork Climate	60.39(17.73)	80.55(16.40)	−7.63	<.001	1.11
% reporting good Safety Climate	58.02(17.71)	81.58(12.53)	−9.88	<.001	1.30
% reporting one or more people in their clinical area often “Intentionally exclude others from the group.”	25.60(14.79)	13.80(13.76)	5.31	<.001	−0.65
% reporting one or more people in their clinical area often “Make comments with sexual, racist, or ethnic slurs.”	8.85(8.20)	3.49(6.21)	4.72	<.001	−0.30
% disagree “I feel burned out from my work.”	58.79(19.71)	68.01(17.60)	−3.18	.002	0.51
% agree “I would like to find a better job.”	18.69(12.75)	13.10(11.36)	2.99	.003	−0.31
% Participated in WalkRounds at least once	13.94(12.25)	24.86(18.66)	−4.50	<.001	0.60
% Received feedback about patient safety risks that were reduced as a result of WalkRounds	14.46(13.16)	30.01(20.62)	−5.84	<.001	0.86

## Discussion

Our brief, 4-item measure of spiritual climate in healthcare appears to be a reliable measure that shows significant variability by healthcare worker role, hospital, and clinical area. The phrase “in this clinical area” provides a clear spiritual climate referent for each item, so it is interesting to note that the small-to-modest effect size for the ANOVA by clinical area translate into moderate to large effect sizes when comparing clinical areas in the top and bottom quartiles of spiritual climate. Clinical areas high on spiritual climate had respondents that reported lower burnout, lower intentions to leave, and lower rates of disruptive behaviors. Respondents reporting positive spiritual climate varied most by clinical area, ranging from 14 to 100%, suggesting that the clinical area environment is largely responsible for determining spiritual climate. Practically speaking, this means that the appropriate level for intervention aimed at affecting spiritual climate may be at the clinical area. Higher scores on spiritual climate were associated with better teamwork and safety climates (and relatively large effect sizes as well). Perhaps the relationships to teamwork and safety are associated with the overall theme of “respect for my views,” found in each of the scales, suggesting convergent validity of our novel spiritual climate scale. In particular, the item “My spiritual views are respected in this clinical area,” is central to the internal consistency of the spiritual climate scale as the reliability would be significantly lower if deleted. The items “In this clinical area, it is difficult to speak up if I perceive a problem with patient care” and “My suggestions about safety would be acted upon if I expressed them to management” from the teamwork climate and safety climate scales, respectively, echo the theme of “respect for my views.” If managers and administrators are able to foster an environment where healthcare workers feel that their spiritual views, an intimate and often taboo topic, are respected and understood, it is easy to imagine that this environment would also enable open conversations about problems with patient care delivery and medical errors. Though we could not test for the effects of type of clinical area, there appeared to be an overrepresentation of emergency departments and pharmacies in the low spiritual climate range, while high spiritual climate scores were common among rehabilitation, home health, and pediatric units. Still there were exceptions to each of these patterns and this should not be over-interpreted.

**Fig. 3:**
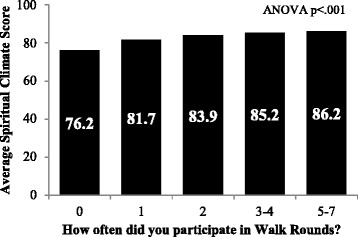
Spiritual Climate and Executive Rounding

Furthermore, the ability of this brief measure of spiritual climate to predict some of the variability in *intention to leave* is encouraging, and is consistent with previous studies [6, 7]. This may be good news for managers and directors working in healthcare, who are struggling to find new and better ways to improve engagement and meaning for their staff. Promoting a respectful spiritual climate may open new doors for them. The link between spiritual climate and participation in executive rounding (Fig. 3), which is a quality improvement initiative, suggests that spiritual climate is sensitive to intervention and that other interventions more targeted at spiritual climate may be effective [12]. Even more than frequency of participating in executive rounding, it was those respondents who reported receiving feedback about actions taken as a result of executive rounding that reported the highest spiritual climate. If receiving feedback about the progress of quality improvement initiatives is associated with stronger spiritual climate scores, then perhaps future interventions could target spiritual climate improvements and discussions (feedback) with healthcare workers. While specific interventions to augment spiritual climate in healthcare workers have not been reported in the literature to date, Grant et al. reported that nurses who attended meetings where spirituality was frequently discussed were much more cognizant of their colleagues’ desire to express and talk openly about spirituality [4]. Spiritual climate was related to teamwork climate in our study. However, unlike teamwork climate, where physicians report higher levels of satisfaction with collaboration norms than nurses, [13, 14] the spiritual climate results showed that managers and nurse managers were more positive than nurses, who in turn reported better spiritual climate than physicians. If the theme of feeling respected and deeply understood undergirds spiritual climate, then our results could be interpreted as managers feeling more understood than nurses, who feel more understood than physicians.

Our study has to be interpreted within the context of its design. We analyzed data from only one health system, which was faith-based, and located largely on the west coast of the U.S. It is unknown whether our findings are generalizable to other regions or to secular health care delivery settings. There is potential for spiritual climate to be useful in the large number of extant faith-based hospitals, but it is not clear how these results would be different in non-faith-based hospitals. Previous investigations in safety culture suggest that teamwork and safety climate mean scores are not significantly different in faith based settings relative to non-faith-based settings [15]. Given the moderate associations between spiritual climate and teamwork and safety climate reported here, this would suggest that spiritual climate scores in secular settings would be comparable to these faith-based settings. Also, we relied upon self-report data from healthcare workers, without any independently observable behaviors or outcomes. However, this is standard practice for culture assessments in health care. Moreover, due to the length and nature of the survey administration, our novel scale to assess spiritual climate was introduced without incorporating previously reported workplace spirituality scales to test for convergent validity. Nevertheless, the psychometric and exploratory results from this large sample are encouraging for a brief scale to be used in subsequent research. For a more comprehensive exploration of spirituality as a scientific construct, please see MacDonald et al. [16]. Despite the abundance of research which has been done to improve our ability to assess and address the spiritual needs of our patients, [17–20] little work has been done to define and intervene on the spiritual needs of healthcare workers. Future research should explore how spiritual climate relates to personal well-being, depression, and burnout and test the responsiveness of spiritual climate to interventions designed to improve it. Our tired, busy, and often overwhelmed healthcare workforce deserves to feel “understood” during their working hours.

## Conclusion

Spiritual climate appears to be a clinical area-specific phenomenon that is internally consistent, and associated with teamwork norms, patient safety norms, disruptive behaviors, burnout and intention to leave. There appears to be a relationship with executive rounding participation, which suggests that spiritual climate may be responsive to intervention and could be a target for initiatives that improve teamwork, safety, and turnover rates.

## Abbreviations

LPN: Licensed practicing nurses
LVN: Licensed vocational nurses
RN: Registered nurse
SAQ: Safety attitudes questionnaire
